# Update: variable implementation of the 2018 UKCGG/UKGTN guidelines for breast cancer gene panel tests offered by UK genetics services

**DOI:** 10.1136/jmedgenet-2020-107529

**Published:** 2021-02-10

**Authors:** Sarah Wedderburn, Stephanie Archer, Marc Tischkowitz, Helen Hanson, UKCGG, Kai Ren Ong

**Affiliations:** 1 Clinical Genetics, NHS Greater Glasgow and Clyde, Glasgow, UK; 2 Department of Public Health and Primary Care, University of Cambridge, Cambridge, UK; 3 Academic Department of Medical Genetics, University of Cambridge, Cambridge, UK; 4 South West Thames Regional Genetic Services, St George's University Hospitals NHS Foundation Trust, London, UK

**Keywords:** genetic testing, genetic counseling

In 2017, the UK Cancer Genetics Group (UKCGG) and UK Genetic Testing Network (UKGTN) held a workshop which led to a consensus for UK cancer gene panel testing.^1^ The agreed breast cancer panel included *BRCA1, BRCA2, PALB2, ATM, CHEK2, PTEN, STK11* and *TP53*. The genes *NBN, BRIP1, BARD1* and *CDH1* were discussed, but excluded from the panel. The agreed ovarian cancer panel included *BRCA1, BRCA2, BRIP1, MLH1, MSH2, MSH6, RAD51C* and *RAD51D*. The agreed genes were included as there is sufficient evidence of a clear association with breast or ovarian cancer predisposition and identifying a pathogenic variant in one of these genes would have clinical implications for cancer management, surveillance or risk reducing surgery. Of note, eligibility criteria for these panels were not addressed at the workshop. During March–May 2020, UKCGG conducted a review of breast cancer panel testing offered in the UK; each UK genetics centre was asked to complete a survey about testing (online supplemental information).

10.1136/jmedgenet-2020-107529.supp1 Supplementary data

**Figure 1:**
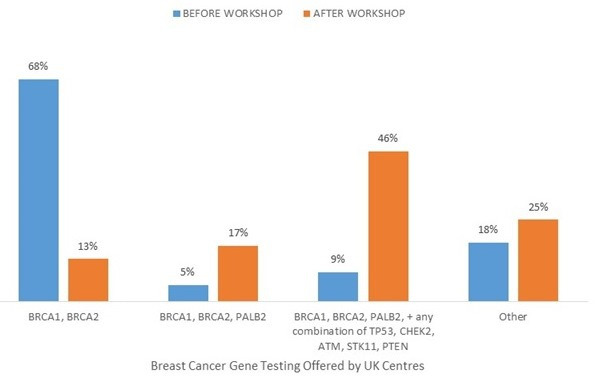
Comparison of what breast cancer gene testing was offered to non-syndromic breast cancer families in 24 UK Genetics Centres, before and after the UKCGG/UKGTN Inherited Cancer Panel workshop (n=24 responses).

There was a 100% response rate from the 24 centres. Figure 1 shows a comparison of testing pre-2018 versus post-2018 workshop. While some inconsistency remains on testing offered, there is a continued trend towards gene panel testing as agreed in 2018. Centres were additionally asked what testing they planned to offer following the introduction of the National Genomic Test Directory, which sets out the genomic tests commissioned by the National Health Service England and corresponding eligibility criteria.^2^ While the first draft of the Test Directory (TD) was published in October 2018 and the current version in August 2020, genomic laboratory hubs are still transitioning to full implementation. The TD recommended a smaller panel consisting of *BRCA1*, *BRCA2* and *PALB2* for inherited breast cancer and isolated non-mucinous epithelial ovarian cancer (Criteria R208) with exclusion of *ATM* and *CHEK2. PTEN, STK11, TP53* and *CDH1* are recommended in specific situations where there are either additional syndromic features, specific pathology or young age of onset (Criteria R212, R213, R215, R216). An ovarian cancer panel as per UKCGG/UKGTN is recommended only where there are two or more cases of ovarian cancer in a family (Criteria R207).^2^ With implementation of the TD, 33% of centres will offer *BRCA1/BRCA2/PALB2,* and any combination of *TP53, CHEK2, ATM, STK11* or *PTEN*, 38% will offer only *BRCA1/BRCA2/PALB2,* and 29% planned to offer an alternative option for inherited breast cancer.

**Figure 2:**
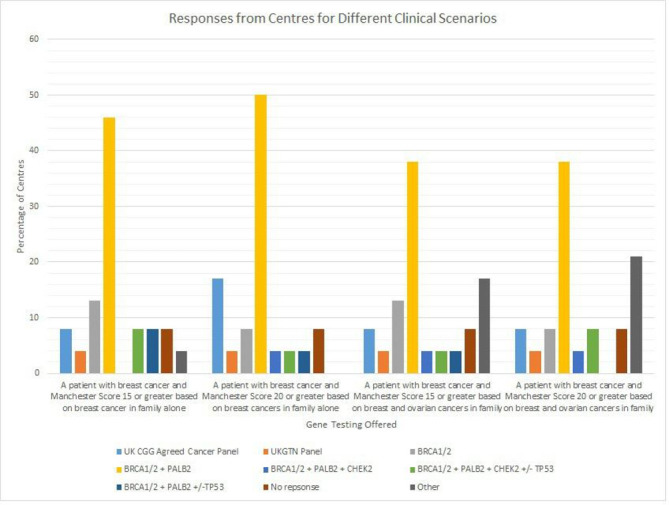
Responses from the 24 UK Genetics centres for different clinical scenarios.

In reality, testing is not proscriptive, as seen in figure 2 which summarises the responses to a variety of case scenarios. Centres are currently using a combination of TD criteria, national and/or local guidance, and the Manchester scoring system^3^ to direct testing decisions. The reasons for these differences are multifaceted and may reflect the recent reconfiguration of genetic laboratory services and the creation of the TD for centres in England which occurred after the 2018 guidelines were published, but has not yet been fully implemented in all centres. There is not a specific directory for the devolved nations, although some centres have chosen to follow the TD.

In summary, it appears that there is a willingness to move towards the 2018 consensus, but the ongoing differences in gene testing offered between centres continues to raise concerns about the current equity of service for patients and their families across the UK. Additionally, the difference in the recommendations from the UKCGG/UKGTN meeting and the TD have resulted in further variation in practice, particularly for the moderate risk breast cancer predisposition genes *ATM* and *CHEK2*. This is largely due to the UKCGG/UKGTN assessing only the appropriate inclusion of genes on a specific panel and not the entry point for testing, which has been specified through the TD. Since the consensus meeting and first draft of the TD, there have been considerable advances in risk estimation for carriers of a pathogenic variant in *ATM* and *CHEK2* through the CanRisk model.^4^ This demonstrates the importance of a responsive TD that can adapt to new information that will impact both inclusion of genes on a specific panel and eligibility for testing. It is hoped that variation will be reduced once full implementation of the National TD takes place and the process for timely amendments to the TD is finalised.
