# Supplementary material for: Update: variable implementation of the 2018 UKCGG/UKGTN guidelines for breast cancer gene panel tests offered by UK genetics services
Source: J Med Genet. 2021 Feb 10;58(8):579–80. doi: 10.1136/jmedgenet-2020-107529 (PMC8327314; doi:10.1136/jmedgenet-2020-107529)
Supplement: Supplementary data [file jmedgenet-2020-107529supp001.pdf]

Survey Questions

Q1 - Which genetics centre are you from?

Q2 - What breast cancer gene testing was offered to non-syndromic breast cancer patients/families in your centre BEFORE the UKCGG/UKGTN Cancer Leads Inherited Cancer Panel workshop (May 2017)?

Q3 - What breast cancer gene testing was offered to non-syndromic breast cancer patients/families in your centre AFTER the UKCGG/UKGTN Cancer Leads Inherited Cancer Panel workshop (May 2017)?

Q4 - What breast cancer gene testing will your centre offer to non-syndromic breast cancer patients/families from April 2020 (i.e. when the test directory goes live)?

Q5 - Is your centre following the test directory criteria R208?

Q6 - If your centre is NOT using the test directory criteria R208 what are you using as guidance for gene testing?

Q7 - What testing would you offer for a case of isolated non-mucinous ovarian cancer?

Q8 - What testing would you offer for a patient with breast cancer and Manchester Score 15 or greater based on breast cancer in family alone?

Q9 - What testing would you offer for a patient with breast cancer and Manchester Score 20 or greater based on breast cancers in family alone?

Q10 - What testing would you offer for a patient with breast cancer and Manchester Score 15 or greater based on breast and ovarian cancers in family?

Q11 - What testing would you offer for a patient with breast cancer and Manchester Score 20 or greater based on breast and ovarian cancers in family?

Q12 - If you have any additional comments, please add them here:

BMJ Publishing Group Limited (BMJ) disclaims all liability and responsibility arising from any reliance placed on this supplemental material which has been supplied by the author(s)
